# Longitudinal volumetric analysis of *in ovo* compartments in chicken eggs using ultra-high-field magnetic resonance imaging

**DOI:** 10.3389/fvets.2024.1450572

**Published:** 2024-12-17

**Authors:** Felix Streckenbach, Hanna Schön, Julia König, Marcus Frank, Inga Langner, Oliver Stachs, Anika Jonitz-Heincke, Sönke Langner, Tobias Lindner, Jana Schätzel

**Affiliations:** ^1^Institute for Diagnostic and Interventional Radiology, Pediatric and Neuroradiology, Rostock University Medical Center, Rostock, Germany; ^2^Faculty of Medicine Carl Gustav Carus, University Center for Orthopedics, Trauma and Plastic Surgery, Technische Universität Dresden, Dresden, Germany; ^3^Department of Diagnostic and Interventional Neuroradiology, School of Medicine and Health, Technical University of Munich, Munich, Germany; ^4^Department of Anesthesia and Intensive Care, University Hospital Rostock, Rostock, Germany; ^5^Medical Biology and Electron Microscopy Centre, Rostock University Medical Center, Rostock, Germany; ^6^Department Life, Light and Matter, University of Rostock, Rostock, Germany; ^7^Department of Orthopedics and Orthopedic Surgery, University Medicine Greifswald, Greifswald, Germany; ^8^Department of Ophthalmology, Rostock University Medical Center, Rostock, Germany; ^9^Research Laboratory for Biomechanics and Implant Technology, Department of Orthopedics, Rostock University Medical Center, Rostock, Germany; ^10^Core Facility Multimodal Small Animal Imaging, Rostock University Medical Center, Rostock, Germany

**Keywords:** *in ovo* cavities, in ovo development, ultra-high field MRI, animal model, chicken embryo

## Abstract

**Introduction:**

The chicken egg, with its *in ovo* compartments, is a widely used and popular animal model in experimental studies. This study aimed to quantify the volumes of the yolk/yolk sac, amniotic fluid, and chicken embryo *in ovo* using non-invasive ultra-high-field magnetic resonance imaging (UHF-MRI).

**Materials and methods:**

In total, 64 chicken eggs were examined using a 7 T UHF-MRI scanner, acquiring T2-weighted anatomical images of the entire egg from developmental day 1 to 16 (D1-D16). Four eggs were scanned each developmental day, and the volumes of the yolk/yolk sac, amniotic fluid, and embryo were quantitatively assessed.

**Results:**

UHF-MRI facilitated the *in ovo* quantitative assessment of the yolk/yolk sac starting from D1 and the embryo from D5 onward. The yolk/yolk sac volume increased from D1 to D6 before progressively decreasing until D14. The amniotic cavity could be detected on D6, with its fluid volume increasing steadily until D14. The embryo’s volume increased consistently throughout the developmental period, reaching its peak at D16.

**Discussion:**

UHF-MRI allows *in vivo* assessment of embryonic development, providing non-invasive, longitudinal insights into the volumes of the yolk/yolk sac, amniotic fluid, and chicken embryo. The investigation method described in this study may provide a standardized model for biomedical research in the developing chicken embryo, supporting various experimental applications.

## Introduction

Ultra-high-field magnetic resonance imaging (UHF-MRI) with magnetic field strength (B_0_) of 7 Tesla (T) and above is an excellent technique for non-invasive and non-destructive imaging. This technique offers excellent soft tissue contrast and high in-plane resolution in the submillimeter range. The higher magnetic field strength enhances resolution and reduces scan time, enabling detailed examination of delicate anatomical structures, such as the embryonic brain or eye, which are often smaller than a few millimeters (1–3). As a non-invasive imaging technique, UHF-MRI allows *in ovo* and *in vivo* studies of chicken embryos throughout their entire developmental period, as demonstrated previously (4–9).

During the 21-day developmental period before hatching, the egg contents and surrounding eggshells provide all necessary resources except oxygen and heat (10, 11). Due to its short developmental period and relatively easy handling, the chicken egg, with its *in ovo* compartments, is a widely used and popular animal model for experimental studies (12–21).

During development, three extraembryonic membranes are formed, including the yolk/yolk sac membrane, the amnion, and the chorioallantoic membrane (CAM) (22, 23). All essential nutrients, including proteins, lipids, carbohydrates, and minerals, are provided by the yolk/yolk sac. The yolk/yolk sac is important for blood cell synthesis, especially erythrocytes. The amnion encloses the embryo and protects it from mechanical and thermal shocks. Before hatching, the embryo ingests the amniotic fluid as a source for water and nutrients. During development, the allantois fuses with the chorion to form the CAM. This heavily vascularized compartment is used as a respiratory organ along the eggshell and is involved in the acid–base balance (24).

Furthermore, this compartment is important for the excretion and storage of waste products from the embryo (25, 26). A key resource for the early growth of the embryo is the albumen, a source of water and proteins (10–12). Parolini et al. decided to inject into the albumen rather than into the yolk because of the amphipathic properties of perfluorooctanesulfonate and its high binding affinity to proteins, such as lipoproteins and albumin (12).

As mentioned above, all compartments play an important role in the embryonic development of the chicken. Therefore, this animal model has been frequently used to evaluate possible impacts on the developmental processes that occur after changes and manipulations of these compartments in ovo, e.g., after injections (12–17).

Over the decades, CAM has been employed to study angiogenesis, tumor growth, metastasizing potential, and cardiovascular development, as the chick embryo is naturally immunodeficient (13, 14). Other studies showed that the chicken embryo is a suitable model to explore the developmental toxicity of various substances and compounds at relevant concentrations for humans (15). Briels et al. injected perfluorooctanesulfonate and its chlorinated polyfluoroalkyl ether sulfonate alternative F53-B, separately and as mixtures, into the yolk sac at the beginning of the developmental period (15).

Petrovová et al. examined agrochemicals’ effects and potential embryotoxicity, including pesticides, by injecting a cholinesterase inhibitor, bendiocarb, in the chicken embryo (16). In 2010, de Siqueira Bueno et al. injected embryotoxic air pollutants into the air sac of the chicken embryo and observed induced abnormalities in embryo–fetal development (17).

All studies have in common that they calculated the doses of the injected substances in relation to the total weight of the egg without focusing on the volumes of the injected compartments. However, this approach does not consider developmental differences in the targeted compartment in relation to the embryo size and growth, thus making an accurate delivery of the tested substances questionable. In addition, some authors assume a uniform distribution of the applied compounds throughout all egg compartments after injecting persistent organic pollutants in the allantois (18), again neglecting the considerable changes in these compartments with respect to embryo nutrition and growth.

Quantifying the volumes of the yolk, yolk sac, and amniotic fluid in avian eggs is essential for establishing a standardized animal model in developmental biology. These measurements provide critical insights into nutrient utilization and key stages of embryonic growth, enabling precise monitoring of developmental patterns. Tracking these volumes also enhances our understanding of physiological processes related to embryonic hydration, nutrition, and overall health. Moreover, such data establishes baseline metrics for normal development, supporting comparative studies and allowing researchers to identify anomalies or assess the effects of experimental interventions (10–17, 22, 23).

Therefore, to improve the injection and achieve more accurate concentrations in future studies, it is important to know the volumes of the compartments during the developmental period. Therefore, this study aimed to quantify the volumes of yolk/yolk sac, amniotic fluid, and the chicken embryo over the entire developmental period *in ovo* and *in vivo* by using non-invasive UHF-MRI.

## Materials and methods

### Animal model

All animals were handled in accordance with the ARVO statement for the Use of Animals in Ophthalmic and Vision Research, and the experiments complied with national legislation for the protection of animals. Sixty-four fertilized chicken eggs (White Leghorn), obtained from a commercial hatchery (Valo BioMedia, Osterholz-Scharmbeck, Germany), were stored at room temperature (20°C) for 3 days prior to the start of incubation. All 64 eggs were simultaneously incubated (Heka-Turbo 168, HEKA, Rietberg, Germany) at optimal conditions: 37.8°C and 60% relative humidity, as recommended by the manufacturer of the incubator. Each day (D1-D16), four eggs were scanned once, respectively, and incubation was terminated. For euthanasia, the chicken eggs were cooled on crushed ice for 60 min in total. Afterward, the eggs were opened following decapitation. None of the embryos hatched. After euthanasia, the yolk sac was extracted, and the volume was measured manually in a water-filled measurement cylinder, as detailed below.

### Ultra-high-field MR imaging

*In vivo* imaging was conducted on a 7 T MRI scanner (BioSpec 70/30, Bruker Biospin MRI GmbH, Ettlingen, Germany) equipped with a BGA-12S HP gradient (bore size of 11 cm) and a circularly polarized volume coil (rat body coil with 112 mm/72 mm outer/inner diameter, Bruker Biospin). First, a fast T2-weighted (T2w) localizer was acquired. Following the localizer, a high-resolution T2w turbo spin-echo sequence (TurboRARE – Rapid Acquisition with Relaxation Enhancement) of the entire egg was performed in coronal planes. Imaging parameters were time of repetition (TR)/time of echo (TE) 3,740/27 ms; matrix size 368 × 368; field of view (FOV) 40 × 40 mm; in-plane resolution 0.150 × 0.150 mm; slice thickness 1.0 mm; no slice gap; time of acquisition (TA), 10:38 min.

Motion artifacts became more pronounced from D8 onward, impairing image quality. Therefore, the eggs were removed from the incubator and placed on crushed ice for at least 20 min to minimize motion inside the egg, and afterward, an MRI was performed. Following that, the eggs were terminated. As demonstrated in different studies (2, 4–6), eggs can typically be returned to the incubator after 40–60 min on crushed ice without adversely affecting development, allowing incubation to continue. This well-established procedure is essential for preventing motion artifacts. Temperature was monitored during the entire scanning time using an MR-compatible thermometer (1,025 T, Monitoring & Gating system, Small Animal Instruments, United States) as described previously (4, 5).

### MR image evaluation

**Figure 1:**
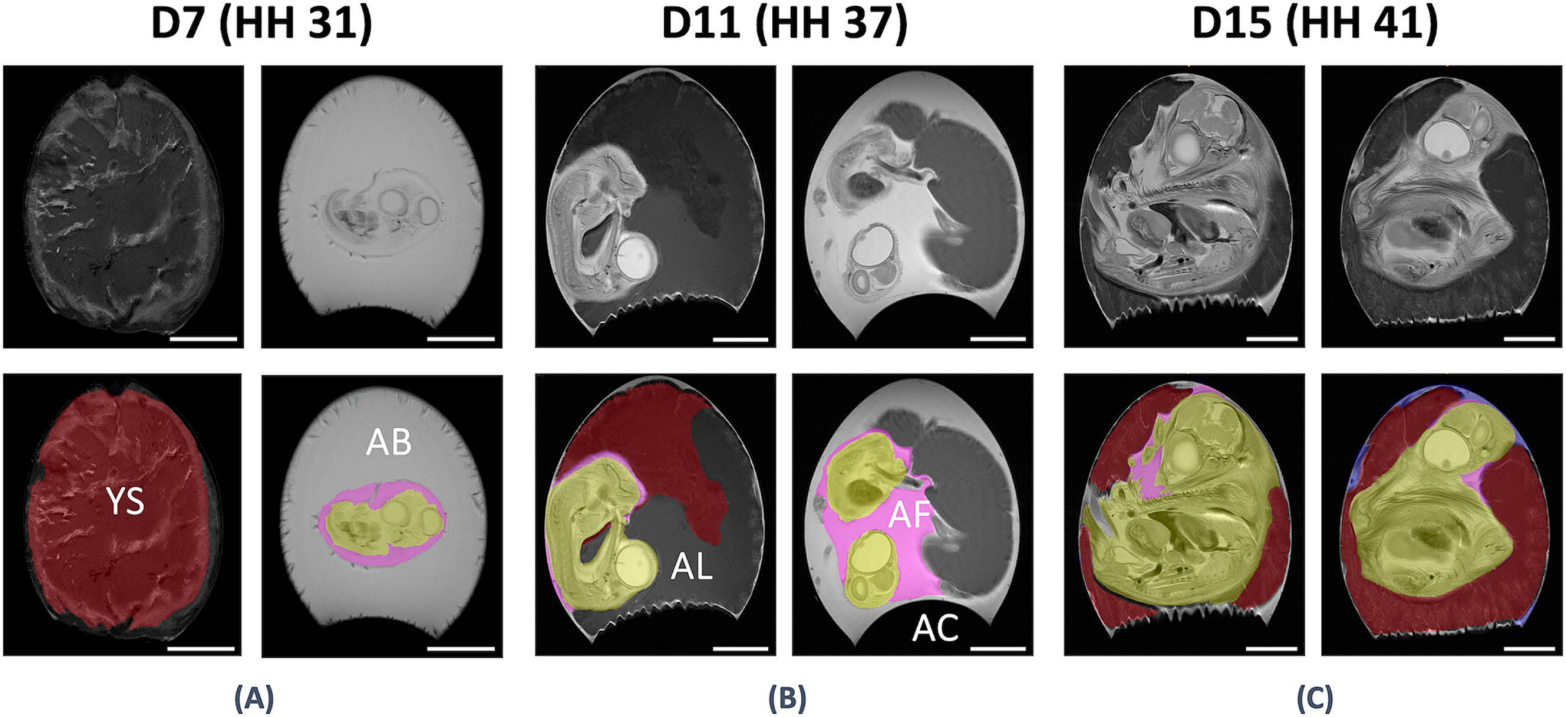
Anatomical evaluation of the in-ovo compartments using UHF-MRI. Anatomical assessment of the in ovo compartments at D7 / HH stage 31 **(A)**, D11 / HH stage 37 **(B)**, and D15 / HH stage 41 **(C)** on coronal T2-weighted UHF-MR images with (bottom) and without (top) segmentation. It was possible to differentiate between the yolk/yolk sac (YS, red), amniotic fluid (AF, pink), albumen (AB), allantois (AL), air chamber (AC), and the chicken embryo (CE) on all respective slides of raw MRI data. Bar ≙ 10 mm.

For further workup, the MR datasets were transferred to an ITK-Snap workstation (Ver. 3.8.0) (27), and contours of the yolk/yolk sac, amniotic cavity with its fluid, and the chicken embryo were manually defined on all respective slides of raw data (see Figure 1).

To evaluate the reproducibility of the analysis, this procedure was repeated three times, and mean values were calculated for each region of interest.

### Evaluation of yolk sac *ex vivo*

After MRI, the embryos were euthanized, and the yolk sac was collected separately. A 100 mL measuring cylinder (EcoLab E-1266, neoLab Migge GmbH, Heidelberg, Germany) was filled with 50 mL of clear water (20° C), and the yolk sac was placed into the cylinder. The volume was determined by measuring the increase in water level within the cylinder. This established procedure for volume determination is referred to as the Archimedean method (28, 29).

### Statistical analysis

Volume values of yolk/yolk sac, amniotic fluid, and the chicken embryo are provided as mean ± standard deviation using IBM® SPSS® Advanced Statistics 22.0. A regression line was determined with an R2 closest to 1 for the amniotic fluid and the chicken embryo. To ascertain whether there is a significant difference between the volumes of the yolk sac *in ovo* and *ex ovo*, a paired t-test was conducted based on the assumption of a *p*-value of <0.05.

## Results

All eggs were successfully incubated. MRI identified changes in inner egg structures starting from D1 and identified changes in the embryo from D5 onward. Quantification of the yolk and, subsequently, the yolk sac was feasible from D1 onward, while the amniotic cavity could be evaluated from D6 onward. All results are shown in Table 1.

**Table 1:** Quantitative evaluation of the volume of the yolk/yolk sac, the amniotic fluid, and the chicken embryo in ml from D1 to D16.

Developmental day/HH stages	Yolk/yolk sac in ml	Amniotic fluid in ml	Embryo in ml
1/5–7	14.7 ± 1.4	–	–
2/11–13	15.4 ± 0.9	–	–
3/18	16.9 ± 2.1	–	–
4/23	19.8 ± 1.0	–	–
5/26	24.6 ± 1.3	–	0.1 ± 0.0
6/29	25.4 ± 1.6	0.1 ± 0.0	0.3 ± 0.1
7/31	24.5 ± 1.7	0.9 ± 0.1	0.5 ± 0.0
8/34	23.5 ± 2.1	2.2 ± 0.5	1.1 ± 0.6
9/35	22.5 ± 2.0	2.6 ± 0.3	1.9 ± 0.5
10/36	20.2 ± 2.8	3.3 ± 0.2	2.7 ± 0.6
11/37	15.2 ± 1.0	3.6 ± 0.3	4.1 ± 0.9
12/38	9.4 ± 1.2	4.0 ± 0.4	4.3 ± 0.3
13/39	6.9 ± 3.1	4.4 ± 1.0	8.6 ± 2.8
14/40	5.0 ± 1.0	5.2 ± 0.9	10.8 ± 1.4
15/41	6.1 ± 1.1	–	11.1 ± 1.7
16/42*	3.9 ± 0.9	–	16.4 ± 1.1

*Incubation was terminated on D16/HH stage 42 to ensure that none of the embryos hatched (Mean ± standard deviation). The average volumes (in ml) of the yolk/yolk sac, amniotic fluid, and embryo were measured for four different eggs at each developmental day (D1–D16) and corresponding HH stages, with standard deviations provided. Quantification of the yolk/yolk sac was feasible from D1 (HH stage 5–7) onward; the embryo was assessed from D5 (HH stage 26) onward, and the amniotic cavity was evaluated starting from D6 (HH stage 29).

### Yolk/yolk sac

**Figure 2:**
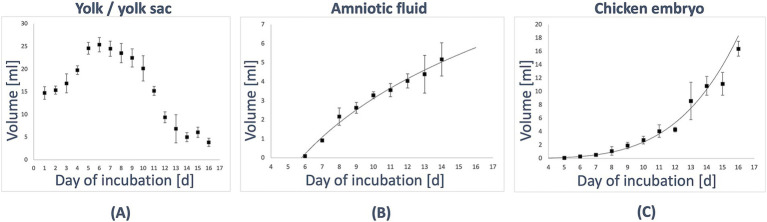
Volumes of the in-ovo compartments during development. **(A)** The yolk/yolk sac was identified on D1 (HH stage 5–7) *in ovo*. Initially, its volume gradually increased from (14.7 ± 1.4) ml on D1 (HH stage 5–7) to (25.4 ± 1.6) ml on D6 (HH stage 29). Subsequently, the volume consistently decreased, reaching (5.0 ± 1.0) ml by D14 (HH stage 40). **(B)** The amniotic cavity was detectable starting on D6 (HH stage 29), with an initial fluid volume of (0.1 ± 0.0) ml. From this point, the volume increased logarithmically (R^2^ = 0.987), reaching (5.2 ± 0.9) ml by D14 (HH stage 40). **(C)** The volume of the embryo increased exponentially (R^2^ = 0.989), starting from (0.1 ± 0.0) ml on D5 (HH stage 26) and growing to (16.4 ± 1.1) ml by D16 (HH stage 42).

First, the volume slowly increased between D1 with (mean ± standard deviation: 14.7 mL ± 1.4 mL) to (25.4 ± 1.6) ml on D6. Afterward, the volume consistently decreased until D14 with (5.0 ± 1.0) ml. The most significant change could be observed between D10 and D12, from a volume of (20.2 ± 2.8) ml to (9.4 ± 1.2) ml. A minor increase of the volume was observed on D15 to (6.1 ± 1.1) ml before it decreased again to (3.9 ± 0.9) ml on D16 (see Table 1; Figure 2A).

**Figure 3:**
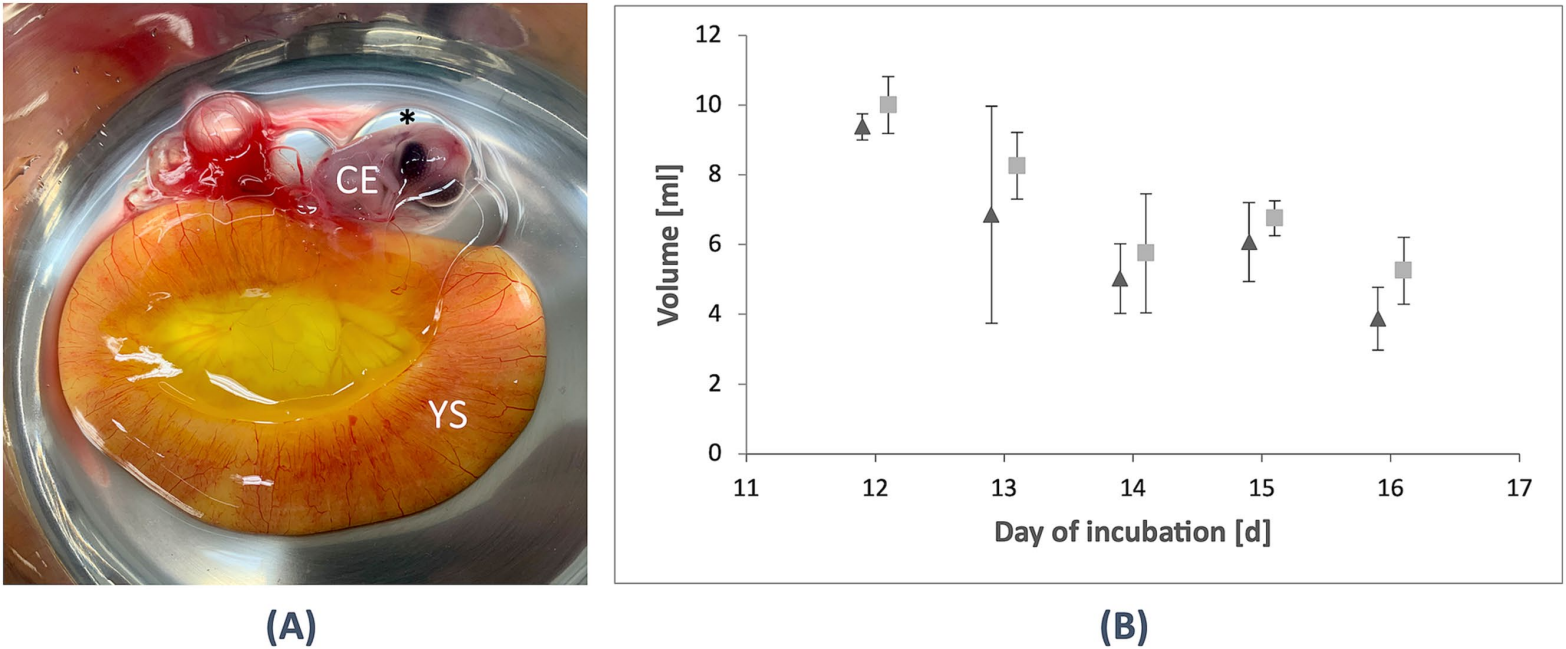
Evaluation of the yolk sac in ovo and ex ovo. **(A)** After MRI, incubation was terminated, and the eggs were opened. From D12 / HH stage 38 onward, it was possible to extract the yolk sac (YS) and observe the chicken embryo (CE) and the amniotic cavity (*). **(B)** A comparison was made between the yolk sac volume in ovo (triangle) and *ex ovo* (rectangle) from D12–16 (HH stage 38–42). While the *in ovo* measurements showed slightly smaller volumes overall, a minor increase in the *ex ovo* volumes was observed, peaking on D15 (HH stage 41) before decreasing again on D16 (HH stage 42). These findings contribute to the anatomical evaluation of *in ovo* compartments using UHF-MRI.

After MRI, the eggs were opened, and it was possible to separate the yolk sac with its thin membrane without damage from D12 onward. The volume was measured on D12 with (10.3 ± 0.5) ml and decreased to (5.8 ± 1.7) ml on D14. Moreover, in the *in ovo* measurement, a minor increase of the volume was observed on D15 to (6.8 ± 0.5) ml before it decreased again to (5.3 ± 1.0) ml on D16 (see Table 2; Figure 3). No significant differences could be observed based on the assumption of a *p*-value of <0.05.

**Table 2:** Volumes of the yolk sac in ovo and ex ovo between D12 / HH stage 38 and D16 / HH stage 42.

Yolk sac in ml			
Developmental day/HH stages	*In ovo*	*Ex ovo*	*p-values*
12/38	9.4 ± 0.4	10.3 ± 0.5	*p* = 0.07
13/39	6.9 ± 3.1	8.3 ± 1.0	*p* = 0.23
14/40	5.0 ± 1.0	5.8 ± 1.7	*p* = 0.27
15/41	6.1 ± 1.1	6.8 ± 0.5	p = 0.19
16/42	3.9 ± 0.9	5.3 ± 1.0	*p* = 0.19

(Mean ± standard deviation) The volumes of the yolk sac determined through MRI imaging (*in ovo*) are shown on the one hand, and on the other hand, in comparison to ex ovo measurement. No significant difference between both methods was found, based on the assumption of a *p*-value of <0.05. *Incubation was terminated on D16/HH stage 42 to ensure that none of the embryos hatched.

### Amniotic cavity

The amniotic cavity could be observed with a fluid volume of (0.1 ± 0.0) ml on D6. Afterward, it increased in a logarithmic way (R2 = 0.987) to (5.2 ± 0.9) ml on D14 (see Table 1; Figure 2B).

### Embryo

The volume of the embryo was first segmented on D5, with measurements indicating an exponential increase (*R*^2^ = 0.989) from (0.1 ± 0.0) ml on D5 to (16.4 ± 1.1) ml on D16. Significant growth changes were observed, particularly between D12 and D13, where the volume increased from (4.3 ± 0.3) ml to (8.6 ± 2.8) ml, and between D15 and D16, where it grew from (11.1 ± 1.7) ml to (16.4 ± 1.1) ml. Detailed data are presented in Table 1; Figure 2C.

## Discussion

In contrast to the morphological and functional longitudinal development of the chicken embryo, investigation of the *in-ovo* development of the compartments is scarce. In 1951, Hamburger and Hamilton (HH) provided 46 chronological stages that allowed the developing chick to be accurately characterized during all embryonic stages but did not focus on the *in-ovo* compartments (19).

To date, there have been several approaches for studying the impact of different injections into the respective embryonic compartments, especially yolk/yolk sacs, amniotic fluid, and chicken embryos (12–18, 20). The amniotic injection route is the most popular because of its fast distribution, as substances may be quickly absorbed orally and through mucosal surfaces (30). Nevertheless, an application through the chorioallantoic membrane or the amniotic cavity may lead to reduced hatchability compared to applications where the yolk sac route or the extraembryonic cavity is used (31). Injection of the air sac is usually made in an early stage of development, while in a later stage, the amniotic route is preferred, as the embryo starts to consume amniotic fluids (32).

The majority of the above-mentioned studies share the approach of calculating the doses of injected substances relative to the total weight of the egg, rather than considering the specific volumes of the injected compartments.

UHF-MRI allowed the quantitative assessment of the yolk/yolk sac starting from D1 and the embryo from D5 onward. The volume of the yolk/yolk sac first increased between D1 and D6, which might have been caused by the water flux from the albumen into the yolk/yolk sac (23). The amniotic cavity could be observed on D6, and its fluid volume increased to D14. In this study, one of the reasons for the growth is the transfer of albumen into the amniotic cavity (21).

There was an increasing volume of the embryo during the observed developmental period. The volumes we obtained in our study are similar in range compared to the few values already known from the literature. In contrast with our study, Adriaensen et al. quantified the volume of the yolk sac, amniotic fluid, and chicken embryo but solely on D11, 13, and 15 using MRI (21). On D11, the volume of amniotic fluid was summarized as about (3.6 ± 0.30) ml in our study and about (3.42 ± 0.25) ml as described by Adriaensen et al., which is quite similar. In this study, the volume of the embryo and the yolk sac is about (16.84 ± 1.13) ml in total at D13. We were able to quantify the volume of the embryo and yolk sac at D13 with about (16.50 ± 2.90) ml, which is in accordance with the results of the study mentioned above. We focused on the same compartments, and it was possible to image and quantify the respective volumes and obtain these measurements longitudinally over the entire developmental period until D16.

In another study by Starck et al., the volume of the yolk/yolk sac of ostrich eggs was evaluated at selected developmental time points, and it was possible to observe an increase of the volume in the first 3 weeks with a following decrease until the end of development after 7 weeks (33). Compared to this study, our measured values of the yolk/yolk sac volume are much smaller because the ostrich egg is much larger than the chicken egg, but the trend of the results is similar. This might be also because the yolk is digested by the embryo. Moreover, our observed increase in the embryo volume over the development period is in accordance with general growth and changes in the embryonic weight as described in the literature (19, 34). Referring to this and to our best knowledge, we found no further information in the literature yet.

To validate consistency between *in ovo* and *ex ovo* measurements, embryos were euthanized after MRI, and the yolk sac was collected separately. We demonstrate an excellent correlation between the MRI-based volumetry and the physical measurements after opening the egg. It has also been observed *in ovo* that a minor increase in the volume of the yolk sac was observed by the physical volumetry before it decreased again but with no significant differences (see Table 2 and Figure 3).

We decided to stop the incubation on D16 to ensure that none of the embryos hatched. Otherwise, we managed to overview nearly the entire developmental period except for 4 days. In addition, most of the above-mentioned studies dealing with *in-ovo* injections focused on the first two-thirds of the developmental period. It remains to be seen whether the measurements of the last 4 days of development are relevant for experimental studies, as they are generally not included in current studies.

Manders et al. blindly targeted five embryonic compartments (amniotic cavity, chicken embryo, allantois, albumen, and yolk/yolk sac) with needles of four different lengths by inoculating methylene blue. Three compartments (air chamber, CAM, and blood vessels) were prepared within sight. The visual inspection after breaking the eggs yielded low success rates (20). The reason might be, on the one hand, the constant change in volume of the different compartments and, on the other hand, the variable positions within the egg. It might even be necessary to perform an MRI before every injection to evaluate the exact position. Therefore, our study would greatly benefit future studies and help obtain more accurate injections and, subsequently, more reliable experimental results.

Our study has some limitations. First, one influence might be the cooling of the eggs to prevent motion artifacts after D8, which could have a minor impact on the volumes of the compartments and structures inside the egg, even if it does not influence embryonic development (35, 36). Secondly, although the in-plane resolution of UHF-MRI is excellent, it was sometimes challenging, due to the slice thickness, to differentiate the small structures inside the egg, e.g., the feathers of the embryo. This limitation could be overcome by acquiring isotopic MRI datasets, but there would be a significant increase in acquisition time.

Due to artifact formation, volumetric analysis of air-filled compartments using MRI is challenging. Therefore, we did not focus on the air chamber, which gradually enlarges during development. Isotropic 3D T2-weighted imaging could be attempted to quantify the air sac in future studies.

Finally, the number of embryos used in this study is small, but we demonstrated, as a proof of principle, that the longitudinal volumetric analysis of the amniotic fluid, yolk/yolk sac, and embryo is feasible using MRI. Due to the small number of eggs, we did not correlate our results with the weight and size of the eggs. This could be part of further studies, as there are different types of chicken eggs regarding size and weight (37).

**Figure 4:**
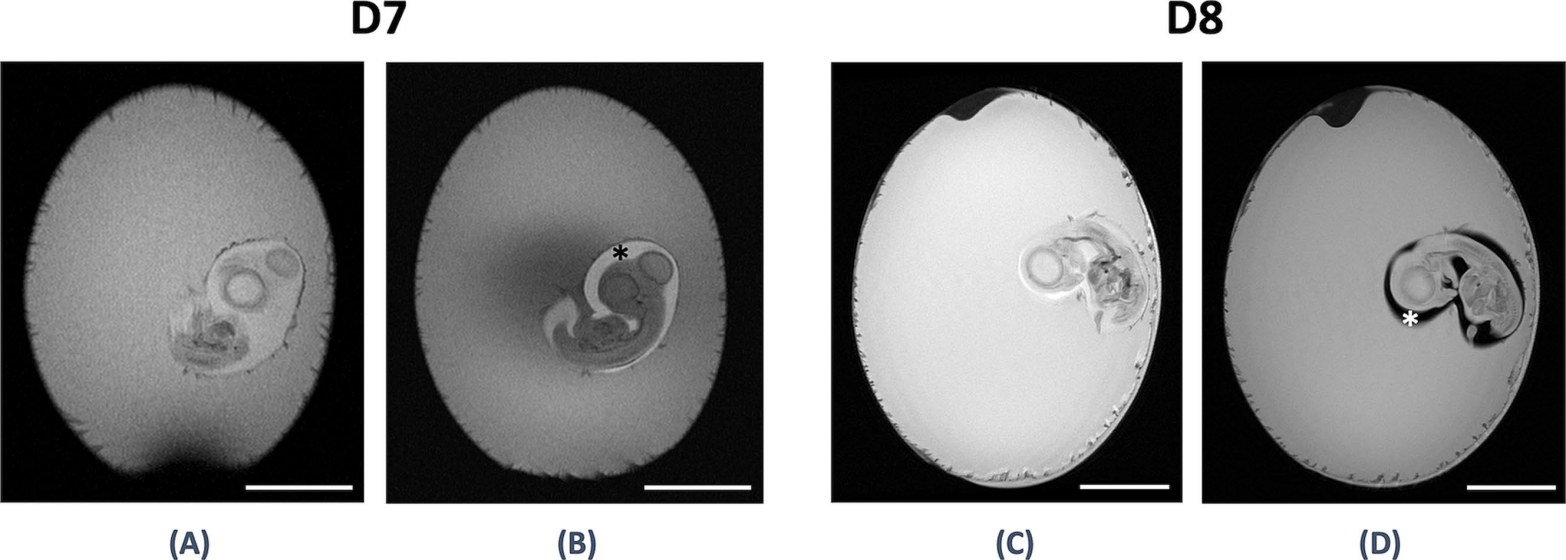
Amniotic cavity before and after injection of Gadovist® and chromium ion solution. T1-FLASH-weighted images acquired before **(A)** and 5 min after **(B)** the injection of 20 μL of Gadovist® (0.0004 mM)-NaCl solution into the amniotic cavity (indicated by a black asterisk) demonstrated a slight increase in the intensity of the amniotic fluid compared to the albumen. Fast spin echo T2-weighted (TurboRARE) MRI images acquired before **(C)** and after **(D)** the injection of 5 μL of a chromium (III) ion solution (11 mM) into the amniotic cavity (indicated by a white asterisk) showed a clear decrease in the intensity of the amniotic fluid surrounding the embryo. The hyperintense fluid seen in **(C)** transitioned to hypointense fluid in **(D)**. Scale bar ≙ 10 mm.

Due to the results of our study, it is possible to conduct targeted injections more precisely and improve the quality of future experiments. For example, it is possible to add Gadobutrol (Gadovist® 1.0 mmoL/mL; Bayer Vital GmbH, Berlin, Germany; 0.0004 mM) as a contrast agent in order to verify the correct injection in the respective compartment using T1-Fast Low-Angle Shot (T1 FLASH) weighted MRI (see Figures 4A,B). It is also possible to evaluate the success of some injections (e.g., chromium ions) without an extra contrast agent and only by the intensity change inside the respective compartment (see Figures 4C,D). MRI could also quantify the compartments’ volumes before and after injections to verify the application site and applicated fluid volume.

In summary, UHF-MRI allows longitudinal volumetric analysis of different compartments of the chicken egg non-invasively and non-destructively, *in vivo* and *in ovo*. This will enable future studies to estimate or even determine the exact individual volume of the respective compartment before an invasive procedure like an injection and to optimize experimental procedures.

## Data Availability

The raw data supporting the conclusions of this article will be made available by the authors, without undue reservation.
